# Koetjapic acid: unveiling its potential as a saviour in the realm of biological and medicinal properties, with a focus on anticancer mechanism of action

**DOI:** 10.1186/s40001-024-01699-6

**Published:** 2024-02-07

**Authors:** Muhammad Armaghan, Khushbukhat Khan, Muhammad Irfan, Amna Hafeez, Sameen Zafar, Zeeshan Javed, Javad Sharifi-Rad, Monica Butnariu, Ioan Sarac, Iulia-Cristina Bagiu, Radu Vasile Bagiu

**Affiliations:** 1 Atta-Ur-Rahman School of Applied Biosciences, National University of Sciences and Technology, Islamabad, Pakistan; 2 Cancer Clinical Research Unit, Trials360 CRO, Lahore, Pakistan; 3 Office for Research Innovation and Commercialization (ORIC), Lahore Garrison University, Sector-C, DHA Phase-VI, Lahore, Pakistan; 4 Facultad de Medicina, Universidad del Azuay, Cuenca, Ecuador; 5 University of Life Sciences ‘‘King Mihai I’’ from Timisoara, 300645 Calea Aradului 119, Timis, Romania; 6 Victor Babes University of Medicine and Pharmacy of Timisoara, Department of Microbiology, Timisoara, Romania; 7 Multidisciplinary Research Center on Antimicrobial Resistance, Timisoara, Romania; 8 Preventive Medicine Study Center, Timisoara, Romania

**Keywords:** Koetjapic acid, Cancer, Apoptosis, Angiogenesis, Natural compounds

## Abstract

Scientists have been compelled to search for alternative treatments due to the increasing prevalence of chemoresistance as well as the agonising and distressing side effects of both chemotherapy and radiation. Plant extracts have been exploited to treat various medical conditions for ages. Considering this fact, the main focus of various recent studies that are being conducted to find new and potent anticancer drugs involves the identification and utilisation of potential therapeutic chemicals present in plant extracts. Koetjapic acid (KJA), which belongs to the family of triterpenes, is primarily isolated from *Sandoricum koetjape*. Ongoing investigations into its therapeutic applications have revealed its tendency to impede the growth and proliferation of cancer cells. Koetjapic acid activates the intrinsic apoptotic pathway and promotes the death of cancer cells. Moreover, it inhibits angiogenesis and the dissemination of tumour (metastasis) by targeting the VEGF signalling cascade. Therefore, this study aims to elucidate the underlying mechanism of anticancer activity of koetjapic acid, providing significant insight into the compound’s potential as an anticancer agent.

## Introduction

Cancer is recognised as a major global health concern and is a leading cause of mortality. In its 2022 report, the American Cancer Society (ACS) have reported 609,360 cancer-related fatalities and roughly 1,918,030 new cancer cases in the USA. The mortality rates for breast and lung cancers increased gradually compared to their incidence in previous years [1]. Conventional treatments such as surgery, radiotherapy, and chemotherapy to cure cancer have been practised for decades without promising results. To mitigate this issue, scientists are exploring and developing novel and targeted treatments such as precision medicine and epigenetic therapies for the treatment of cancer [2].

These targeted therapies are often combined with conventional treatments such as surgery and chemotherapy and can be invasive and painful. Therefore, we focus more on naturally occurring compounds that have proven to be highly beneficial in treatments against cancer. Triterpenes, a class of phytochemicals, have previously been indicated to possess strong anticancer properties against certain cancers, including colon carcinoma [3].

Koetjapic acid (KJA) is present in a tropical fruit, santol (*Sandoricum koetjape*), and is a Seco-A ring oleanane triterpene. This terpenoid-rich medicinal plant grows mainly in Malaysia and Cambodia. X-ray diffraction and NMR studies of its structure showed it to be orthorhombic with nine quaternary carbon atoms [4]. It has been shown that KJA possesses various bioactivities, including anti-inflammatory, antibacterial, antiangiogenic, and DNA polymerase inhibitory properties. It can activate intrinsic and extrinsic caspases to induce apoptosis in colon cancer cell lines. It can also cause nuclear content condensation, DNA fragmentation, and changes in the potential of mitochondrial membrane. KJA has also been found to modulate various cellular signalling pathways. KJA can downregulates signalling pathways such as HIF-1α and MAP/JNK/ERK pathways, while it can also upregulate some other signalling pathways, including NF-κB pathway [5]. However, detailed literature indicating the KJA mechanism of action, and its clinical use is unavailable. Therefore, this study aims to discuss the KJA mechanism of action in cancers and its potential clinical use. This study highlights the therapeutic and chemopreventive potential of KJA, laying the foundation for designing future studies on KJA targeting cancer hallmarks such as angiogenesis, apoptosis evasion, and metastasis.

### Review methodology

Electronic databases such as PubMed, Science Direct, and the Google Scholar search engine were employed to search the studies related to KJA and its derivatives’ anticancer properties. MeSH terms “Koetjapic acid”, “Cancer, Koetjapic acid”, “Koetjapic acid, anticancer properties”, “Koetjapic acid, clinical properties”, and “Koetjapic acid, plant sources”. Papers discussing pharmacology, koetjapic acid's subcellular analysis, preclinical cancer models, and clinical trials involving the koetjapic acid efficacy assessment were included. This review only considered those studies that were published in the English language, while those studies that were published in other languages were not included.

### General characterisation of koetjapic acid

KJA with the IUPAC NAME “(3*S*,4*aR*,6*aR*,7*S*,8*S*,10*aR*,10*bS*,12*aS*)-7-(2-carboxyethyl)-3,7,10*a*,10*b*,12*a*-pentamethyl-8-prop-1-en-2-yl-2,4,4*a*,6,6*a*,8,9,10,11,12-decahedron-1*H*-chrysene-3-carboxylic acid” is a terpenoid, which has been identified and isolated from *S. koetjape* extracts, which is a medicinal plant in Meliaceae family, indigenous to Cambodia, Malaysia, and Laos. The molecular formula of KJA is C30H46O4, with a molecular weight of 470.7 g/mol. KJA is known to be a seco-rig compound because of the presence of an H-atom at each terminal group of the compound [6, 7].

**Fig. 1:**
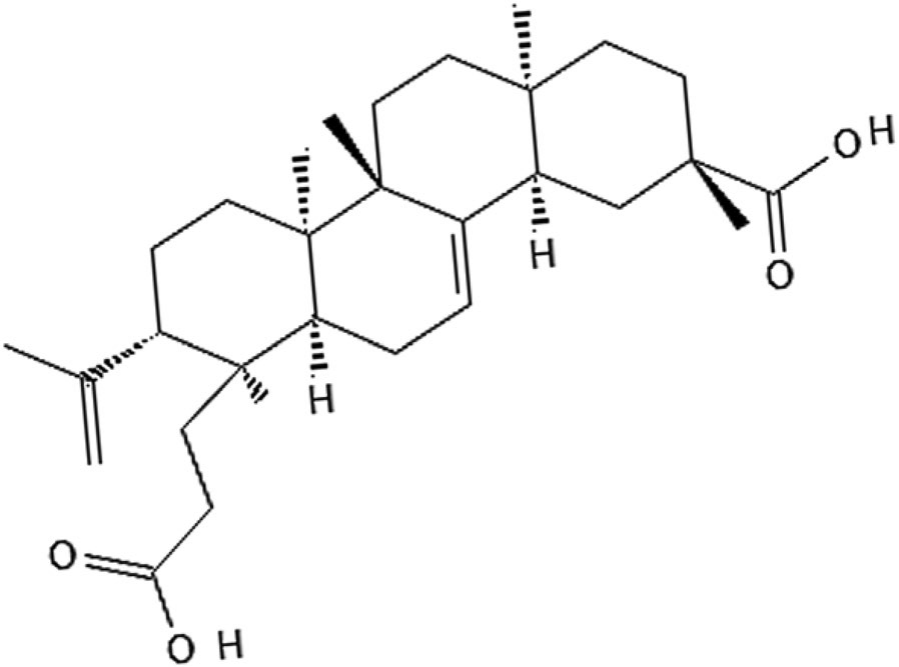
Representation of the chemical structure of koetjapic acid

The KJA is constituted of a tetra-rings system in a fused configuration. The rings A/B/C and D form conjugation as C/D, A/B, and B/C forms by adopting trans, cis and E- E-confirmations, respectively. These rings of KJA attain various configurations as ring-A has a configuration form between the half chair and envelope, and ring-B has an enveloped form configuration. In contrast, C and D's last two rings have attained chair-like confirmation. The weak intramolecular H-bonds have been observed in the molecule of KJA, and each molecule of KJA is linked to form dimers by a couple of pairs of intermolecular Hydrogen-bonds O–H^….^O, which were found to be piled up along C-axis [6]. The chemical structure of KJA is shown in Fig. 1.

### Plant sources of koetjapic acid

Koetjapic acid was first identified in the stem bark of *Sandoricum koetjape*, which is also its primary source. The traditional medicinal plant *Sandoricum koetjape* is a member of the *Meliaceae* family, a rich source of koetjapic acid. Southeast Asian nations, notably Malaysia, Indonesia and the Philippines, are its native habitats. It is referred to as santol locally in Malaysia. The medium-sized tree produces eatable fruit. In Indonesia, traditional healers utilise a decoction from the plant's bark to cure leucorrhoea and colic. Malaysia's bark’s aqueous extract is also customarily taken as a tonic after childbirth. Similarly, the bark and leaves have been applied topically for medical purposes [4].

KJA has also been extracted from *Dillenia* species. Around a hundred species of woody shrubs and trees of the genus *Dillenia* can be found in the subtropics and seasonal tropics of Australia, Asia, and Oceania [8]. Traditional healers use plants from this family to treat various ailments, particularly related to gastrointestinal system such as diarrhoea which is treated using extracts from this plant [9]. Some species of Dillenia also produce fruits. A tiny tree native to Indonesia (Sulawesi) called *Dillenia* s*errata* Thunb yields a palatable, sweetish-acid fruit. The locals traditionally use this plant's fruit, stem bark, and wood. The golden fruit can be eaten straight up and is used to acidify food. Meanwhile, locals use the stem bark decoction of this plant for oral treatment of blood vomiting [10].

### Bioavailability and pharmacokinetics of koetjapic acid

It has been found that koetjapic acid displays a variety of pharmacological characteristics, such as anti-inflammatory activity by preventing the generation of PGE2, anti-proliferative activity in the breast cancer MCF-7 cells, inhibition of DNA polymerase, and antibacterial activity [4]. The plant may have anti-inflammatory benefits in several body organs, and in vitro tests of chemical extracts from santol stems revealed that they have anticancer qualities. Santol seed extracts contain insecticidal qualities [11]. Using column chromatography, the KJA may be separated from the stem bark of *S. koetjape*, and its cytotoxic action has been investigated [7].

It has been discovered that KJA has limited solubility in water and other cell culture-allowed solvents, such as dimethyl sulfoxide, which presents a challenge for future research and its therapeutic application. A chemical alteration of KJA into its salt form, potassium koetjapate, has been reported to improve solubility. Potassium koetjapate (KKA) displayed in vitro efficacy due to its improved water solubility [12]. The potassium koetjapate (KKA) pharmacokinetic properties in rats have been successfully determined using the HPLC method. The outcomes showed that oral administration of KKA resulted in rapid absorption into the rat circulation.

Additionally, the antiangiogenic experiments showed a notable increase in KKA's efficacy versus its parent molecule, koetjapic acid. By preventing VEGF expression and endothelial activities, KKA reduced angiogenesis. KKA’s improved solubility and bioavailability are probably responsible for its improved bioefficacy. In order to establish KKA as an efficient treatment drug against illnesses associated with angiogenesis, additional preclinical and clinical investigations are required [12].

### Metabolism of koetjapic acid

The metabolism studies of koetjapic acid remain elusive. In general, enzymes by the body mediate various actions, including reduction, oxidation, and hydrolysis, in the metabolism of natural chemicals. The functional groups found in the structure of koetjapic would determine their specific metabolic destiny. Several different enzymes frequently participate in the metabolism of xenobiotics, including natural compounds such as triterpenoids [13]. However, the exact process of metabolising koetjapic acid is still unknown.

### The anti-bacterial and anti-viral potential of koetjapic acid

A study investigating the antimicrobial potential of koetjapic acid, extracted from *Dysoxylum hainanense*, unfolded as a significant contribution to microbiology. This study shed light on koetjapic acid's remarkable antibacterial properties, particularly its efficacy against Gram-positive bacteria. Impressively, it surpassed the performance of the positive control, magnolol, underlining its potent antibacterial activity. Koetjapic acid emerged as a promising antimicrobial agent, displaying inhibitory effects against specific Gram-positive bacterial strains such as *S. epidermidis* and *B. subtilis*, with minimum inhibitory concentrations (MIC) of 12.5 µg/mL and 6.25 µg/mL, respectively. However, a noteworthy limitation was its selectivity, as koetjapic acid exclusively exhibited antibacterial activity against Gram-positive bacteria, remaining ineffective against Gram-negative bacteria and fungi.

Nevertheless, these findings emphasised koetjapic acid's potential to combat Gram-positive bacterial infections, underscoring its significance in the ongoing pursuit of novel antimicrobial agents [14]. A few studies have shown that KJA possesses antimicrobial activities [14–16]. The activity of KJA, along with other triterpenoids, has been investigated for its antibacterial role against a broad range of bacteria with the help of a modified micro-titre plate assay. It was shown that KJA contained the most significant antibacterial activity as it inhibits the growth of *S. aureus*, MRSA, and *P. aeruginosa* with the MIC value between 3.125 and 12.50 μg/mL [17]. Extracts of KJA from the upper parts of *M. undata* and various other OA derivatives have shown significant antibacterial activity against S. aureus ATCC33591 with MIC of 12.5 mg/L and 6.25 mg/L against MSSA. Moreover, the epi-KJA also demonstrated antimicrobial activity against MSSA and MRSA strains with MIC of 3.25 mg/L and 6.25 mg/L, respectively [18, 19].

KJA has also been reported to inhibit the Epstein–Barr virus early-antigen (EBV-EA) activation in Raji cells, demonstrating the anti-proliferative role. The ichthyotoxic potential of KJA was also determined [20], which revealed that the compound induced the cytotoxicity of tumour cell lines by inhibiting the activity of DNA polymerase β (β-pol). β-pol is a key player in the repair process of damaged DNA nucleotides through the base excision repair (BER) system [21–23]. Studies have shown that β-pol knockout mice are excessively sensitive towards DNA methylation through compounds like methane sulfonate. Residues in the 8-kDa domain of the polymerase have been known to interact with KJA, a natural inhibitor of β-pol. The NMR analysis of KJA-induced DNA polymerase inhibition revealed that this compound was bound with β-pol with a higher affinity of 11Μm. KJA was found to be attached to 8-kDa domain of β-pol [21].

### Mechanism of antitumor action of koetjapic acid

**Fig. 2:**
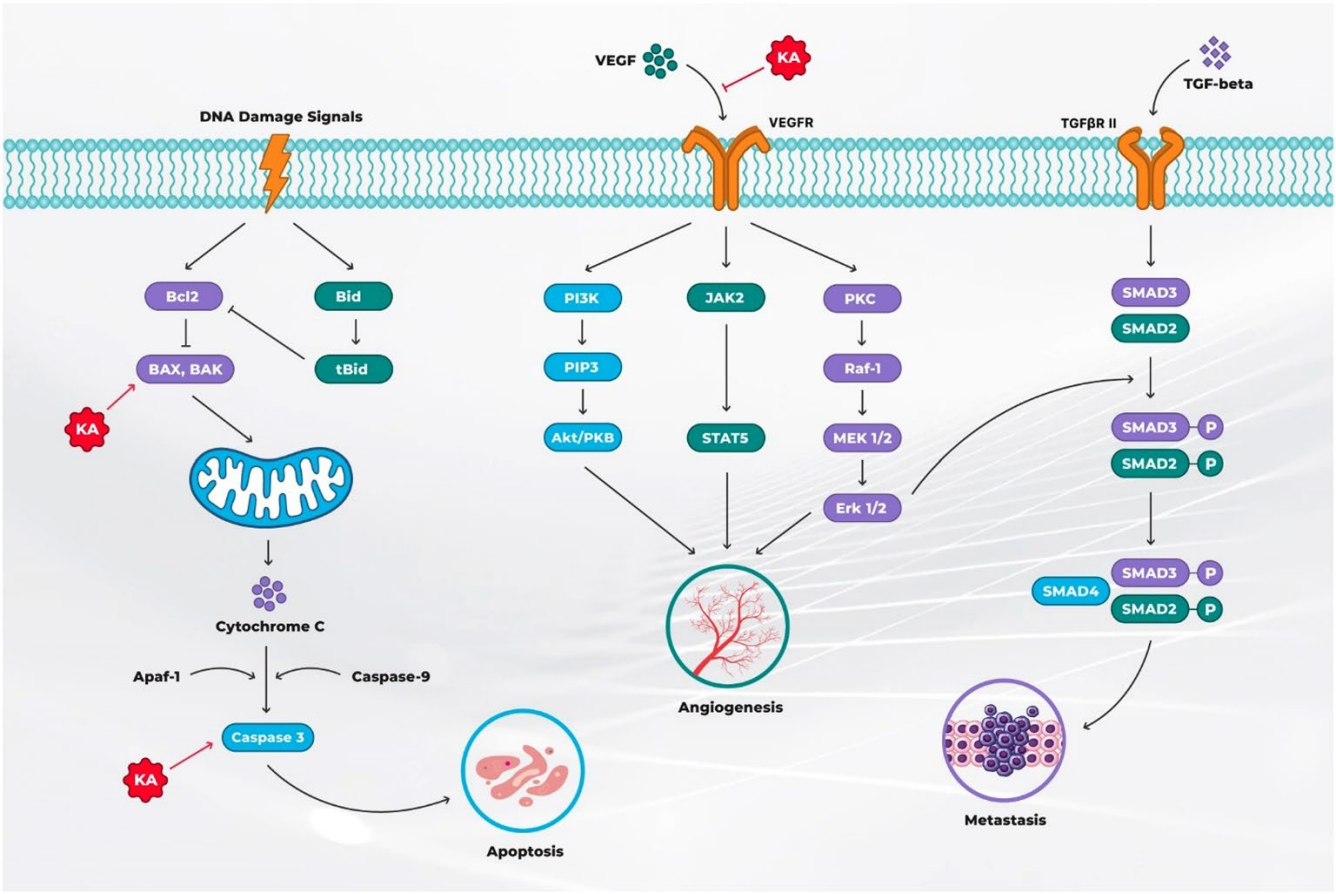
Koetjapic acid molecular mechanism in curbing carcinogenesis. KA promotes the signalling through the cell’s apoptotic pathways in cancer cells by promoting the activity of BAX, BAK, and Caspase 3. It also inhibits the VEGF signalling pathway and targets angiogenesis. KA koetjapic acid, PIP3 phosphatidylinositol (3,4,5)-trisphosphate, PKB protein kinase B, PKC protein kinase C, SMAD suppressor of mothers against decapentaplegic, TGF transforming growth factor, VEGF vascular endothelial growth factors

KJA obtained from the stem bark of an evergreen plant, *S. koetjape,* is a seco-A-ring oleanane triterpene with antitumor properties against different human cancers [24, 25]. Besides anticancer activities, KJA also has an antibacterial [26], anti-inflammatory [27], and ichthyotoxic and chemopreventive properties [28] (Fig. 2). So far, literature indicated that KJA promotes cancer cell death and causes angiogenesis regression, as discussed below.

#### Effects of koetjapic acid in cancer cell lines

The anticancer role of KJA against different pathways involved in breast cancer has been investigated in several investigations. KJA targets angiogenesis, apoptosis, and metastasis in breast cancer. The antimetastatic and apoptotic potential of KJA was investigated on breast cancer MCF-7 cells. KJA inhibits breast cancer proliferation and induces programmed cell death. Further experiments investigated the underlying mechanism behind the induction of apoptosis, which revealed that KJA induces apoptosis mainly by activating caspases 3/7, inducing DNA fragmentation, nucleus deformation and loss of mitochondrial membrane potential, which are the main events involved in the apoptotic pathway [29]. The same study also confirmed that KJA inhibits the migration, invasion, as well as colony formation ability of MCF-7 cells. Experiments based on wound healing assay demonstrated that KJA at 15 μg/ml concentration inhibited the migration of breast cancer cells. Moreover, at a higher concentration of 40 μg/ml, it also obstructed the clonogenicity of MCF-7, thus inhibiting the metastasis in breast cancer [30] (Table 1).

**Table 1:** Effects of koetjapic acid on cancer cell lines

Study	Cell line/model	Dosage/duration	Outcome	Mechanism of action
Study 1	Breast cancer (MCF-7)	KJA: IC_50_ 68 μg/mL	Apoptosis, cytotoxicity, anti-proliferative, anti-metastatic	Activation of caspases, mitochondrial apoptotic pathway, inhibition of invasion and migration
Study 2	Colorectal carcinoma (HCT 116)	KA: IC_50_18.88 µg/ml	Apoptosis, cytotoxicity	Activation of caspases, DNA fragmentation, nuclear condensation
Study 3	Colorectal carcinoma (HCT 116)	Potassium koetjapate: higher solubility and cytotoxicity, LD_50_ > 2000 mg/kg in rats	Apoptosis, enhanced cytotoxicity, safety for oral administration	Activation of caspases, mitochondrial membrane disruption, enhanced solubility

Koetjapic acid (KA) was extracted from the *Sandoricum koetjape* plant, and its selective anticancer potential against colorectal carcinoma was discovered. It was clear that KA had potential as a systemic anticancer drug to treat colorectal cancer, but its therapeutic value was limited by its incredibly low solubility. The main goal of this study was to overcome this solubility issue while assessing the anticancer effectiveness of potassium koetjapate, a recently synthesised derivative, in human colorectal cancer cells. The researchers investigated several strategies to address the solubility issue, including the development of an inclusion complex with (2-hydroxypropyl)-β-cyclodextrin and solid dispersions utilising carboxymethyl cellulose, polyvinylpyrrolidone and sodium lauryl sulphate. Furthermore, they created potassium koetjapate, a salt form of KA [31]. The main results of this study showed potassium koetjapate to be the best formulation among those evaluated, showing noticeably more excellent solubility and increased cytotoxicity against HCT 116 colorectal cancer cells. Potassium koetjapate's increased effectiveness was related to its capacity to cause apoptosis, as shown by the target cells' nuclear condensation and disruption of the mitochondrial membrane potential. Surprisingly, the study also demonstrated that potassium koetjapate was safe for rats to consume after oral treatment, with an LD_50_ of more than 2000 mg/kg [31].

The anticancer potential of KA was brought into sharp relief in a different but related study, notably in terms of its capacity to target HCT 116 colorectal carcinoma cells through the complex apoptotic pathways. The research uncovered a striking selectivity of KA, as it exhibited significantly greater cytotoxicity towards HCT 116 cells, with an IC_50_ value of 18.88 µg/ml, compared to MDA-MB231, CCD-18Co, and Hep G2 cell lines. In HCT 116 cells treated with KA, increased levels of major caspases (caspase-8, − 9, and caspase-3/7) were found, demonstrating the participation of both extrinsic and intrinsic apoptotic pathways [5]. This result was consistent with other studies that suggested caspase-8 alone might not be sufficient to induce efficient cell death; instead, it may need to engage with the intrinsic apoptotic pathway by starting to disrupt the mitochondrial membrane, which then caused the release of cytochrome c. Additionally, KA had a dose-dependent effect on HCT 116 cells, causing DNA fragmentation, nuclear fragmentation, and chromatin condensation that resulted in the production of apoptotic bodies. These combined results highlight the complex yet effective apoptotic mechanisms by which KA exerts its anticancer effects, highlighting its potential as a treatment for colorectal cancer.

KJA is a natural inhibitor of metastatic neoplasm. Studies have shown this compound induces apoptosis in MCF-7 cell lines [32]. KJA cytotoxic influence in MCF-7 cells (IC_50_ 68 μg/mL) is also reported. It was also shown that KJA was involved in the mitochondrial apoptotic pathway. Furthermore, KJA also inhibited the invasion and migration of MCF7 cells at IC-50 15 μg/mL, indicating that KJA contains significant anti-proliferative and anti-metastatic effects. In vitro screening of KJA revealed the cytotoxic effect of this compound on the OSCC cell line by inducing apoptosis with IC-50 of 18.88 μg/mL [33]. Further studies showed that it induces apoptosis in colorectal cancer HCT116 cells through inducing apoptotic pathways (extrinsic and intrinsic), mediated by DNA fragmentation, mitochondrial membrane disruption, and nuclear condensation [31, 34]. Further studies have shown that KJA downregulates the expression of various proteins involved in essential cellular pathways, including NF-κB, MAPK, Wnt, HIF-1α, JAK/STAT, Akt/mTOR, and C-MYC pathways [5, 32, 35].

#### Koetjapic acid-mediated angiogenesis inhibition

Several compounds of natural origin have shown their anticancer potential by inducing inhibition of angiogenesis. One of the important hallmarks of cancer is angiogenesis, which can be targeted as a treatment strategy for different cancers [36]. A recent study demonstrated that a naturally occurring compound galloylquinic acid suppressed the expression of VEGF and induced cytotoxicity in MCF-7 breast cancer cells [37]. Moreover, a study also revealed that galloylquinic acid along with doxorubicin, a chemotherapeutic agent, downregulated various oncogenic pathways by remarkably reducing the levels of Notch-1, VEGF, TNF-α, IL-6, cyclin D1, and NFkB, while increasing the activity of capsase-3 in tumour tissues [38]. Other natural compounds such as curcumin, triterpenoids, celastrol, tocotrienols have shown promising tendency to inhibit angiogenesis promoting proteins such as VEGF/VEGFR, FGF/FGFR, HIF. The angiogenesis is a sequential process that includes endothelial cell activation followed by the basement membrane disintegration, cell migration, formation and elongation of vessel branches, vasodilation, and remodelling [39]. The angiogenesis process depends on the level of both pro and anti-angiogenic factors [40]. Angiogenesis is induced when the expression of angiogenic factors such as VEGF increases compared to antiangiogenic factors such as endostatin [41]. VEGF is a primary pro-angiogenic molecule that stimulates the process of neovascularisation and angiogenesis [42]. Natural inhibitors of VEGF from medicinal plant sources tend to impart their effects by employing various mechanisms such as modulation of VEGF activating signalling pathways, inhibition of VEGF expression [43]. While the chemotherapeutic agents tend to inhibit cancer cell proliferation but they induce systemic cytotoxicity and have low bioavailability [44]. Hence, investigating natural compounds with high bioavailability and anti-angiogenic activity can help in the development of new and more potent anticancer therapeutics.

KJA exerts its antitumor activities by targeting different pathways involved in cancer pathogenesis. KJA has been shown to possess antiangiogenic properties. The antiangiogenic properties of KJA can be associated with its targeting of angiogenesis cascades. It exerts its antiangiogenic effects by inhibiting the formation of new blood cells, migration and differentiation of endothelial cells and expression of VEGF factor, which is the main positive angiogenic factor [45, 46]. The metastasis is another significant event in carcinogenesis, which consists of multiple steps including cell survival, colony formation, migration and invasion. The migration is important for tumour cells to leave their primary site and access a new secondary site through the circulatory system. KJA can potentially cause the retardation of migration and invasion of cancer cells at the sub-cytotoxic level. Moreover, it also inhibits the formation of colonies, another metastatic event [47].

#### Koetjapic acid-induced cancer cell apoptosis

The induction of apoptosis is another way KJA exerts its anticancer activities [48]. KJA has been found to induce apoptosis by activating executioner caspases 3/7, which further cleaves cellular proteins like β-catenin, E-cadherin and cytokeratin-18, leading to the disassembly and disintegration of the cell. It is involved in causing chromatin condensation, DNA and nuclear fragmentation in a dose-dependent manner, leading to the formation of an apoptotic body [36, 49]. KJA also initiates the mitochondrial pathway of apoptosis by activating pro-apoptotic members of Bcl2 family proteins such as Bax and Bak, which leads to loss of membrane potential and, in turn, causes the activation of caspase 3 by releasing cytochrome c [48]. The above studies indicate that KJA can potentially reduce the proliferation and migration of tumour cells and be a safe treatment option for different types of cancers.

### Potential biotechnological studies on in vitro cultures

The extracts of the S. koetjape plant contain various properties of low-cytotoxicity, anti-inflammatory, anti-cancer, anti-bacterial, DNA-polymerase inhibition activity, and ichthyotoxicity [3]. Various studies have reported a potent anti-vasculogenic role of KJA on colorectal cancer cell lines. However, the underlying mechanism is still unknown. The cytotoxic effect of KJA has been identified against various human and murine cell lines. KJA has also shown a chemopreventive role against human cell lines [5].

Nassar et al. have shown that treating KJA on Human Umbilical Vein Endothelial Cells (HUVECs) demonstrates its anti-angiogenic activity. KJA has been shown to have low-cytotoxicity against HUVECs with IC-50 of 40.97 μg/ml, where KJA was found to inhibit the vasculogenesis by alleviating the expression of VEGF, migration of endothelial cells as well as differentiation [3, 4, 11, 50, 51]. In another study, the pharmacokinetics of KJA were studied, and the anti-angiogenic role was investigated in EA.hy926 cell lines. The results showed that KJA resulted in the inhibition of migration, differentiation, and altered expression of VEGF at the IC-50 of 18.4 μM, causing the suppression of angiogenesis. Moreover, KJA was also found to significantly inhibit the formation of micro-vessels in the aorta of rats with the same IC-50 value [12, 19, 52, 53].

KJA is also well regarded as a negative modulator of the production of prostaglandins. PGE2 is derived from arachidonic acid through COX-2, an essential part of the proinflammatory pathway. It was elucidated in a study that KJA significantly inhibited the activity of PGE2 with the IC-50 value of 1.05 μM. Moreover, KJA was also found to reduce the expression of COX-2 enzyme, but phospholipase A2 can also be a target for inhibiting the production of PGE2 [54]. KJA inhibited the inflammatory response when administered orally (50 mg/kg) to the rat model of the paw oedema [27]. The compound was also found to inactivate the COX-2 activity by potentially binding to its active site, as several in silico molecular docking analyses showed. In another study, KJA was found to be slightly more effective on the COX-1 isozyme than the COX-2 isozyme with the IC-50 values of 19.24 and 12.22 at 50 μg for both enzymes, respectively [55]. A recent study revealed that when human blood samples were pre-treated with KJA, they significantly exhibited a strong inhibition of COX-2 at the concentration of 50 μM. Furthermore, the expression of proinflammatory IL-6 and IL-1B is also inhibited by KJA at IC-50 5.02 μM and 4.52 μM, respectively. KJA's ability to target proinflammatory cytokines highlights its potential application as an anti-inflammatory agent [56].

The remarkable potential of koetjapic acid in cancer research justifies additional investigation through in vivo investigations. These in vivo studies could shed important light on the substance's efficacy and safety as a possible cancer therapy, given its strong in vitro anticancer activities. Animal models, such as xenograft or patient-derived tumour models, can be used to evaluate KA's capacity to prevent tumour growth and metastasis. Studies should also determine the best dosage, administration method, and treatment length to maximise anticancer efficacy while minimising adverse effects. In order to determine KA's safety and potential for clinical translation, it will also be essential to assess its pharmacokinetics and toxicity profile in animal models. A deeper comprehension of the compound's mechanisms of action might result from investigating the effect of the substance on important signalling pathways and molecular targets in a living organism. Koetjapic acid's full therapeutic potential in cancer treatment may be unlocked via in vivo research, opening the door for upcoming clinical trials and prospective clinical applications.

### Current medical applications of koetjapic acid

The creation or discovery of non-cytotoxic agents with antiangiogenic and anti-neoplastic properties has recently received increased attention. The adverse effects of conventional chemotherapy medications are reduced by this therapeutic strategy [57]. Endothelial cell activation, basement membrane breakdown, endothelial cell migration, endothelial cell proliferation, vessel lengthening, vessel branching, vasodilatation, formation of the basement membrane, acquisition of pericyte, and remodelling are some of the processes involved in angiogenesis [58]. For several years, cancer colonies have gone undiscovered and inactive. However, under specific conditions, the tumour cells change into an angiogenic phenotype, stimulate the growth of new blood vessels, and then begin to spread to other areas. When pro-angiogenic factors like VEGF outnumber anti-angiogenic factors like endostatin, the angiogenic switch occurs [59]. Therefore, excessive VEGF synthesis may be the cause of the angiogenic switch. Novel non-cytotoxic chemicals or extracts from natural products that exhibit antiangiogenic effects have been the subject of several investigations [60]. The antibacterial, anti-inflammatory, antitumor, and DNA polymerase inhibitory activities of KJA have been discovered. A study conducted to evaluate the antiangiogenic effects of KJA confirmed that KJA stopped the growth of new blood vessels in rat aortic ring explants [3]. However, it was observed that it was not due to the cytotoxic effects of KJA but maybe due to the interruption of one of the many angiogenic cascades. The effects of KJA on three critical stages of angiogenesis, namely endothelial cell migration, endothelial cell differentiation, and VEGF production, were examined to understand the angiogenesis inhibitory mechanisms of KJA better. KJA considerably slows down all of these processes.

The main growth factor that initiates the angiogenesis event is thought to be VEGF. It is in charge of initiating several angiogenesis cascade events, including cell survival, migration, and proliferation. The extracellular matrix and vascular basement membrane are broken down throughout the angiogenesis cascade, allowing endothelial cells to move into the perivascular space in the direction of angiogenic stimuli [61]. Nitric oxide (NO) is a relaxing component generated from endothelium that promotes endothelial cell migration. Numerous studies have demonstrated that VEGF stimulates endothelial NO production and that eNOS is downstream of VEGF [62]. The capacity of KJA to prevent the migration of endothelial cells may be related to its ability to prevent the formation of VEGF, which in turn prevents the signalling of nitric oxide. Endothelial cells undergo differentiation after migrating into the perivascular space, which changes their morphologies and makes it easier for the cells to adhere together to create a lumen [63]. Such tube-like structures were prevented from forming by KJA in a dose-dependent manner. The activation of VEGFR-1 is necessary for the differentiation of endothelial cells. The considerable reduction in VEGF expression caused by KA may contribute to the activation of VEGFR-1 and the subsequent reduction in the endothelial cell differentiation [64]. By a large amount, KJA hindered the vascularisation of the chick embryo [3]. Since KJA blocks several essential angiogenesis-related pathways, it may effectively treat diseases like cancer linked to angiogenesis.

By releasing inflammatory mediators that cause phagocytes to engulf the noxious stimuli and remove injured cells, inflammation is a crucial component of the body's instinctive defence against invaders and restores the structure and physiological function of living tissues [65]. An acute inflammation may be thought of as therapeutic in nature, but the unsatisfactory resolution of responses can lead to a chronic condition, which may then turn toxic and cause tissue damage and function loss [66]. The crucial mediators of inflammatory reactions, prostaglandins (PGs), are produced by the cyclooxygenase (COX) enzymes using arachidonic acid as a substrate. The pathogenesis of inflammation is significantly influenced by these important enzymes [67]. COX1 is constitutively produced in all cells in contrast to COX2, whose production is induced in response to injury, tissue damage or inflammation by proinflammatory cytokines. Compounds that target COX2 as a treatment for inflammation are preferential to those that target COX1, as they may reduce the risks of peptic ulcer and may have reduced gastrointestinal side effects [67]. The in vivo rat model of acute paw oedema caused by carrageenan is frequently used to assess plant extracts' ability to reduce inflammation and identify its chemical components. The release of several inflammatory mediators, including histamine, serotonin, and bradykinin, as well as the biosynthesis of PGs and nitric oxide, produced by inducible COX-2 isoform and nitric oxide synthase (iNOS), respectively, are all part of the well-defined oedema response caused by carrageenan. A study to assess KA's impact on paw oedema found that it had a mild anti-inflammatory effect [55].

In HepG2 cells, KJA has been shown to reduce the production of COXs, inducible nitric oxide synthase (iNOS), and several inflammatory mediators. Additionally, it has been claimed to have strong anti-inflammatory properties by blocking the synthesis of prostaglandin E2 (PGE2) in a human blood assay triggered by lipopolysaccharide. This manner of action might be connected to the suppression of COX2 enzyme activity, which prevents the manufacture of prostaglandins. According to phytochemical studies, the anti-inflammatory effects of KJA may be related to the inhibition of prostaglandin synthesis via the suppression of COX2 expression more so than COX1 expression, which may cause a large reduction in oedema development. The results of the current investigation might support the use of KJA in managing inflammatory disorders [55].

Research was conducted to understand the ability of KJA, isolated from D. serrate, to prevent the PGE2 synthesis in human whole blood. PGE2 production caused by lipopolysaccharide (LPS) has been quantified as an indicator of the activity of cyclooxygenase-2 (COX-2) in blood cells such as monocytes [54]. It is possible to express the reduction in PGE2 synthesis in human whole blood as a reduction in COX-2 enzymatic activity or COX-2 protein expression. The efficacy of KJA to prevent PGE2 in LPS-induced human whole blood was examined. It was found that LPS-induced generation of PGE2 could be inhibited by KJA isolated from D. serrate [54]. The human body produces PGE3 in large quantities and regulates vital biological processes, including immunological, metabolic, neural, and reproductive processes [68]. Although PGE2 has inherent roles, activating the cyclooxygenase-2 (COX-2) pathway of PGE2 is known to be a proinflammatory mediator linked to symptoms like redness, swelling, and pain. Therefore, it would be expected that inhibiting PGE2 production would have analgesic, antipyretic, and anti-inflammatory effects.

### Nano-delivery aspects of koetjapic acid

Nanotechnology involves using small nanomolecular structures to treat cancer either to kill the cancer cells directly or to deliver therapeutic medications at specific tumour sites [69]. Nanomaterial-based treatment for cancer has demonstrated advantages over conventional cancer treatment, particularly in targeted medication administration. Compared to free pharmaceuticals, targeted drug delivery using nanoparticles has led to lower toxic effects, decreased degradation of the drug, improved drug capacity, and longer half-life of drugs [70]. Targeted drug delivery methods based on nanoparticles have advanced recently, incorporating passive and active targeting. Small molecules are used in active targeting systems, whereas passive targeting is accomplished by improving the retention and permeability of nanoparticles [71].

In comparison to traditional chemical therapeutics, nanotechnology-based drug systems exhibit greater bioavailability, increased selectivity, less cytotoxicity, and superior drug loading capacity. So far, a number of nanoparticles have been created for the treatment of cancer with the use of nanotechnology, as well as the advancements in the field of oncology. However, only a small number of drug delivery methods based on nanoparticles have been studied and used in clinical settings [72]. Specific Phyto molecules have been combined with nanoparticle technology in a number of experiments to combat cancer. In several trials, the therapeutic efficacy of the anticancer medicine was further enhanced by conjugating these nanoparticles with specific antibodies for the targeted delivery of drugs. Targeting cancer cells that express particular antigens can increase target-site specificity and less toxicity of these nanoparticle complexes [73, 74].

The nano-delivery aspects of koetjapic acid for cancer treatment remain unexplored. However, few studies have reported the nanotherapeutic effects of koetjapic acid in some other diseases [35]. Koetjapic acid nanoparticles were synthesised and demonstrated antibacterial potential against streptococcus species [75]. A recent study demonstrated that nanoparticles containing koetjapic acid extracts were synthesised and tested on the gingivitis rat models. It was revealed that the nanoformulation of koetjapic acid at a concentration of 2.4% decreased the number of streptococcus colonies after administration [76].

### Koetjapic acid side effects

Koetjapic acid has not been the subject of much research, so details about its adverse effects may be limited. Plant-based substances frequently engage in intricate bodily interactions that might have some adverse effects. The lack of extensive research on koetjapic acid means that specific information about its side effects may be limited. Several studies that have evaluated the anticancer potential of this compound have reported that koetjapic acid did not induce any adverse effect on the normal cells as well as the animal model [12, 33]. However, extensive research is required to confirm and validate the possible side effects of koetjapic acid.

## Conclusion

A triterpene, KJA, targets cancer survival by activating the mitochondrial apoptotic pathway, leading to cell death. Furthermore, KJA also inhibits angiogenesis and, eventually, cancer metastasis. However, the mechanism of action of KJA has not been fully explored. So far, studies have highlighted the contribution of KJA in inhibiting VEGF signalling. Further investigations must be done to understand the KJA’s role in inhibiting hypoxia genes. Studies also show that KJA inhibits the metastasis of cancer cells, but no investigations have been performed to unravel the mechanism of KJA in suppressing cancer. KJA has the potential to be used as a chemopreventive as well as a therapeutic agent for cancers. Therefore, future research must focus on understanding its pharmacological properties and methods to improve its clinical applications. Designing KJA-based therapy will allow physicians to use less painful therapeutic approaches for cancer patients.

## Data Availability

Not applicable.

## Abbreviations

ACS: The American Cancer Society
Bcl-2: B-cell leukemia/lymphoma 2 protein
BER: Base excision repair system
COX2: Cyclooxygenase-2
CRC: Colorectal cancer
EBV-EA: Epstein–Barr virus early-antigen
EMT: Epithelial to mesenchymal transition
eNOS: Endothelial NO· synthase
ERK: Extracellular signal-regulated kinase
ESCC: Esophageal squamous cell carcinoma
HCC: Hepatocellular carcinoma
HER2: Human epidermal growth factor receptor 2
HIF1α: Hypoxia Inducible Factor 1 Subunit Alpha
HUVEC: Human umbelical vein endothelial cells
JNK: C-Jun N-terminal kinases
KKA: Potassium koetjapate
LAC: Lung adenocarcinomas
MAPK: Mitogen-activated protein kinase
MCL-1: Induced myeloid leukemia cell differentiation protein
miR: MicroRNA
MMP: Matrix-metalloproteinases
NSCLC: Non-small cell lung cancer
PGE2: Prostaglandin E2
PEP: Phosphoenolpyruvate
PI3K: Phosphatidylinositol 3-kinase
PIM1: Proto-oncogene serine/threonine-protein kinase
PKB/AKT: Protein kinase B
PKM: Pyruvate kinase M
PNS: Panax notoginseng saponins
PR: Progesterone receptor
TGFβ 1: Transforming growth factor beta 1
TNBC: Triple-negative breast cancer
VEGF: Vascular endothelial growth factors
